# circ_NOTCH3 Functions as a Protooncogene Competing With miR-205-5p, Modulating KLF12 Expression and Promoting the Development and Progression of Basal-Like Breast Carcinoma

**DOI:** 10.3389/fonc.2020.602694

**Published:** 2021-01-20

**Authors:** Bing Guan, Qing Li, Hui-Zhen Zhang, Hai-Sheng Yang

**Affiliations:** ^1^ Department of Pathology, Shanghai 6^th^ People’s Hospital Jinshan Branch, Shanghai, China; ^2^ Department of Pathology, Shanghai Pudong New Area People’s Hospital, Shanghai, China; ^3^ Department of Pathology, Shanghai 6^th^ People’s Hospital, Shanghai, China

**Keywords:** basal-like breast carcinoma, circ_NOTCH3, circ_RNA, miR-205-5p, KLF12, protooncogene, progression, biomarker

## Abstract

Breast cancer is the most common type of cancer diagnosed among women, and basal-like breast carcinoma (BLBC) has been associated with a more aggressive histology, poorer prognosis, and non-responsiveness to hormone therapy. In the present study, the role and molecular mechanism of circular (circ)_NOTCH3 in the development and progression for BLBC was identified. circ_RNAs array was used to screen the ectopic expression of hsa_circ_0109177 (circ_NOTCH3) in BLBC. RT-qPCR was conducted to evaluate the circ_NOTCH3 expression in BLBC tissues and paired normal tissues, as well as related cell lines. Cell function changes were analyzed following circ_NOTCH3 or micro (mi)RNA overexpression or co-expression. Bioinformatics analysis and dual-luciferase reporter assay were performed to predict and verify the binding sites between circ_NOTCH3 and miRNAs. Gene expression changes were assessed using western blotting. circ_NOTCH3 had a significantly higher expression in BLBC tissues and cell lines. The upregulation of circ_NOTCH3 promoted the proliferation, migration, invasion and inhibited the apoptosis for BLBC cells. The opposite results were observed following miR-205-5p overexpression. However, the co-expression of circ_NOTCH3 and miR-205-5p resulted in those restoration. circ_NOTCH3 is capable of binding to miR-205-5p, and upregulating its target gene KLF12, which can be downregulated by miR-205-5p overexpression and restored by the co-expression of circ_NOTCH3 and miR205-5p. circ_NOTCH3, being an protooncogene and a powerful biomarker, can function as a sponge, compete with miR-205-5p, modulate KLF12 expression, and promote the development and progression of BLBC.

## Introduction

Breast cancer is the most common type of cancer diagnosed among women in the USA, and the second leading cause of cancer mortality among US women after lung cancer (1, 2). Breast cancer incidence rates in women have been seeing a slight annual rise (by ~0.3% per year) since 2004 in US (1, 2). The 5-year relative survival rate for female breast cancer (90%) is highest in the USA (1). In 2015, breast cancer was the most commonly diagnosed cancer among Chinese women and the 6th leading cause of cancer mortality among Chinese women (3). Breast cancer alone is expected to account for 15% of all new cancers in Chinese women (3). Breast cancer has been a major public health problem worldwide (1–3).

Breast cancer presents as a heterogeneous disease, not only from a clinical and histological perspective, but also from the view of genetic expression; in addition, the intrinsic subtype classification (4) of basal-like breast carcinoma (BLBC) has been associated with a more aggressive histology, poorer prognosis and non-responsiveness to hormone therapy (5–7).

Covalently closed circular (circ)_RNA molecules were originally found in viroid (8) and hepatitis delta virus (9). For a long time, circular RNAs were considered a by-product of splicing errors and lacking biological function (10). However, high-throughput sequencing has identified thousands of circ_RNAs from back-spliced exons in multiple human cell lines (11–13), and it was suggested to function as micro (mi)RNA sponges (12, 14). There is a diversity of circ_RNAs in biological systems. circ_RNAs can be produced by the direct ligation of 5’ and 3’ ends of linear RNAs, or by back-splicing (15). They do not have 5’ caps or poly-A tail structures and are highly stable (16). They have unique properties, including the potential for rolling circle amplification of RNA, ability to rearrange the order of genomic information, protection from exonucleases and constraints on RNA folding (15). They can function as templates for viroid and viral replication, as intermediates in RNA processing reactions, as regulators of transcription in cis, as snoRNAs, and as miRNA sponges (15). circ_RNAs affect human disease, such as changes in the level of ciRS-7/CDR1as (the miR-7 circ_RNA sponge), and alter the levels of miR-7 target genes (both endogenous and reporter) (12, 14, 17). circ_RNAs also play a pivotal role in the pathogenesis and development of a diversity of cancers, such as liver (18, 19), colon (20, 21), gastric (22, 23), lung (24, 25), kidney cancer (26, 27) and endometrial cancer (28, 29).

miR-205-5p was previously identified as a tumor suppressor gene, which directly targeted downstream protooncogene KLF12 and was involved in the progression of BLBC cells (30, 31). What’s more, further circ_RNA array and bioinformatics analysis revealed that circ_NOTCH3 might function as a potential sponge for miR-205-5p. However, direct experimental evidence and detailed mechanisms supporting this model are still lacking. The aim of the present study was to explore circ_NOTCH3 by functioning as a sponge for miR-205-5p modulating KLF12 and its role in the development and progression of BLBC.

## Materials and Methods

### Clinical Specimens

Ten fresh samples of BLBC and paired normal breast tissues which adjacent to tumor from same patient were collected from patients who underwent surgery between January 2017 and December 2018 in the Shanghai 6^th^ People’s Hospital Jinshan Branch and never received preoperative radiotherapy or chemotherapy. The samples were immediately snap-frozen in liquid nitrogen and stored at −80°C for DNA/RNA extraction. Hematoxylin and eosin and immunohistochemistry sections were reviewed and histologically confirmed by two independent pathologists, according to the standard of estrogen receptor (ER), progesterone receptor (PR), human epidermal growth factor receptor 2 (HER2-), epidermal growth factor receptor (EGFR+) and/or cytokeratin 5/6+ (6, 7). The research protocol was officially approved by the Shanghai 6^th^ People’s Hospital Jinshan Branch Medical Ethics Committee, and informed consent was obtained at the time of sample collection.

### Cell Culture

A human BLBC MDA-MB-468 cell line was obtained from the Cell Bank of the Chinese Academy of Sciences and cultured in L-15 medium supplemented with 1% streptomycin and penicillin, and 10% fetal bovine serum (FBS), which were placed in an incubator with 100% air and 37°C for maintenance culture. A human mammary epithelial MCF-10A cell line was obtained from Shanghai Zhong Qiao Xin Zhou Biotechnology Co., Ltd. and cultured in ZQ-1311 special medium, which was then placed in a 5% CO_2_, 37°C incubator for maintenance culture.

### Circ_RNA Array

Total cellular RNA was isolated by TRIzol (Thermo Fisher Scientific, Inc.). Following the removal of linear RNA and rRNA, cDNA was synthesized and amplified and followed by sample labeling using a labeling kit (Aksomics). Next, the labeling samples were mixed pairwise and hybridized with Human circ_RNA Array V2.0 (Aksomics). Finally, the array was scanned and data was extracted for further analysis.

### Plasmid Construction and Transfection

The human cDNA sequences of circ_NOTCH3 were cloned into a PUC57 vector (Fenghuishenwu). MDA-MB-468 cells were transfected with small interfering (si)RNA-circ_NOTCH3, pcd-circ_NOTCH3 expression vector, circ_NOTCH3+mimics-NC, miR-205-5p+mimics-NC and their matched negative controls (Shanghai GenePharma Co., Ltd.) using Lipofectamine 2000 (Thermo Fisher Scientific, Inc.), according to the manufacturer’s instructions.

### Cell Counting Kit-8 (CCK-8) Assay

MDA-MB-468 cells were seeded into 96-well plates at a density of 1x10^4^ cells/ml. Following incubation at 37°C overnight, the cells were transfected with pcd-NC, pcd-circ_NOTCH3, pcd-NC+mimics-NC, pcd-circ_NOTCH3+mimics-NC, pcd-NC+miR-205-5p or pcd-circ_NOTCH3+miR-205-5p. A total of 10 μm CCK-8 solution (Beyotime Institute of Biotechnology) was added into each well following incubation for 0, 24, 48, 72 and 96 h. Following 1 h of incubation, the optical density value of each well was measured at 450 nm using a microplate reader.

### Transwell Assay

A Transwell assay was performed to evaluate the migration and invasion of tumor cells. The cell migration experiment was carried out in an 0.8-μm 24-well chamber (FALCON). The cell invasion experiment was carried out in a BioCoat™ Matrigel^®^ 24-well chamber with 0.8 μm (BioCoat). Medium (700 μl) containing 10% FBS was added into the lower chamber, and 500 μl cell suspension (cell concentration, 2x10^5^/ml) was added into the upper chamber. The cells were further incubated for 24 h in a CO_2_ incubator. Media were then aspirated, and cells in the upper chamber were removed with a cotton swab and fixed with formaldehyde for 30 min at room temperature. The lower side of the chamber membrane was stained with crystal violet for 30 min at room temperature. The polycarbonate film from each chamber was then excised, attached to a slide, covered with a cover slide and sealed with neutral gum. The number of cells was counted in random visual fields under a microscope, and statistical analysis was then performed.

### Flow Cytometry

An apoptosis assay was conducted using Annexin V-fluorescein isothiocyanate (FITC) kit (Beyotime Institute of Biotechnology). A total of 10^5^ resuspended cells were centrifuged at 1,000 x g for 5 min and the supernatant was discarded. Annexin V-FITC binding solution (195 µl) was added and slightly resuspended the cells. Next, 5 µl Annexin V-FITC and 10 µl propidium iodide was added, mixed and incubated for 10–20 min in the dark at room temperature. Apoptosis was measured by flow cytometry (FACSVerse™; BD Biosciences). The data were analyzed using CellQuest software.

### RT-qPCR

Total cellular RNA was extracted using TRIzol (Thermo Fisher Scientific, Inc.), dissolved in RNase-free water, and reverse-transcribed to cDNA using a Reverse Transcription kit (Thermo Fisher Scientific, Inc.). Subsequently, RTqPCR was conducted to amplify the target gene using 2x Master Mix (Roche Diagnostics) and the gene relative expression was normalized to that of GAPDH using the 2^-ΔΔCt^ method. The detailed sequences of the primers used in the present study are presented in in **Table 1**.

**Table 1:** Primer sequences used in this study.

Gene	Sequences (5’ to 3’)
circ-NOTCH3	
Forward	TCTGGTTCCCTGAGGGCTTCT
Reverse	GTCAATCTCCAGCATTACTACCGA
SiRNA-circ-NOTCH3	
Forward	UAAAGCUCGGUAGAAUGCTT
Reverse	GCAUUACUACCGAGCUUUATT
miR-205-5p mimics	
Forward	UCCUUCAUUCCACCGGAGUCUG
Reverse	GACUCCGGUGGAAUGAAGGAUU
KLF12	
Forward	TGGCAAAGCACAAATGGACC
Reverse	CCCTTGATACTGGGGACGGA
siRNA-NC	
Forward	UUCUCCGAACGUGUCACGUTT
Reverse	ACGUGACACGUUCGGAGAATT
Mimics NC	
Forward	UUCUCCGAACGUGUCACGUTT
Reverse	ACGUGACACGUUCGGAGAATT
GAPDH	
Forward	AGAAGGCTGGGGCTCATT
Reverse	TGCTAAGCAGTTGGTGGTG
circ_NOTCH3 wild type	
Forward	CTCTGGTTCCCTGAGGG
circ_NOTCH3 mutant type	
Forward	GATTCTGTCCCACTGCA

Circ, circular; mi, micro; si, small interfering.

### Western Blotting

MDA-MB-468 cells were lysed in radio-immunoprecipitation assay buffer supplemented with protease inhibitors (Thermo Fisher Scientific, Inc.). The proteins were separated by 10% sodium dodecyl sulphate-polyacrylamide gel and then electro-transferred to a polyvinylidene fluoride membrane, followed by blocking with 5% non-fat milk dissolved in Tris-buffered salin Tween 20 (TBST) at room temperature for 3 h. The membranes were incubated with primary antibodies against KLF12 (dilution, 1:1,000; ProteinTech Group, Inc.) and GAPDH (dilution, 1:1,000; ProteinTech Group, Inc.) at 4°C overnight. Following washing 3 times with TBST buffer, the membranes were incubated with horseradish peroxidase-conjugated goat-anti-rabbit secondary antibody (dilution, 1:5,000; Abcam) for 2 h. The protein bands were washed 3 times with TBST buffer and visualized using an enhanced chemiluminescence system (Thermo Fisher Scientific, Inc.).

### Dual-Luciferase Reporter Assay

Luciferase reporter assay was conducted to evaluate the miR-205-5p binding site within the 3’ untranslated region (UTR) of circ_NOTCH3. HEK293T cells were seeded into 48-well plates, and the wild/mutated-type psi-CHECK2 dual-luciferase vectors containing miR-205-5p binding site on circ_NOTCH3 3’-UTR were co-transfected with miR-205-5p mimics or scramble control into the HEK293T cells using Lipofectamine 2000 (Thermo Fisher Scientific, Inc.). After 48 h, the samples were measured for luciferase activity using a Dual-Luciferase Reporter Assay System (Promega Corporation). The relative luciferase signal was represented by the normalization of firefly luciferase to that of *Renilla*.

### Statistical Analysis

All experiments were repeated at least in triplicate. Values are presented as the mean ± SD. Paired t-test and two-way ANOVA were used to analyze the statistical significance between two and multiple groups, respectively. All analysis was conducted with SPSS 21.0 software (International Business Machines Corp). P<0.05 and P<0.01 were considered to indicate a statistically significant difference.

## Results

### circ_RNA Array Expression Analysis

**Figure 1:**
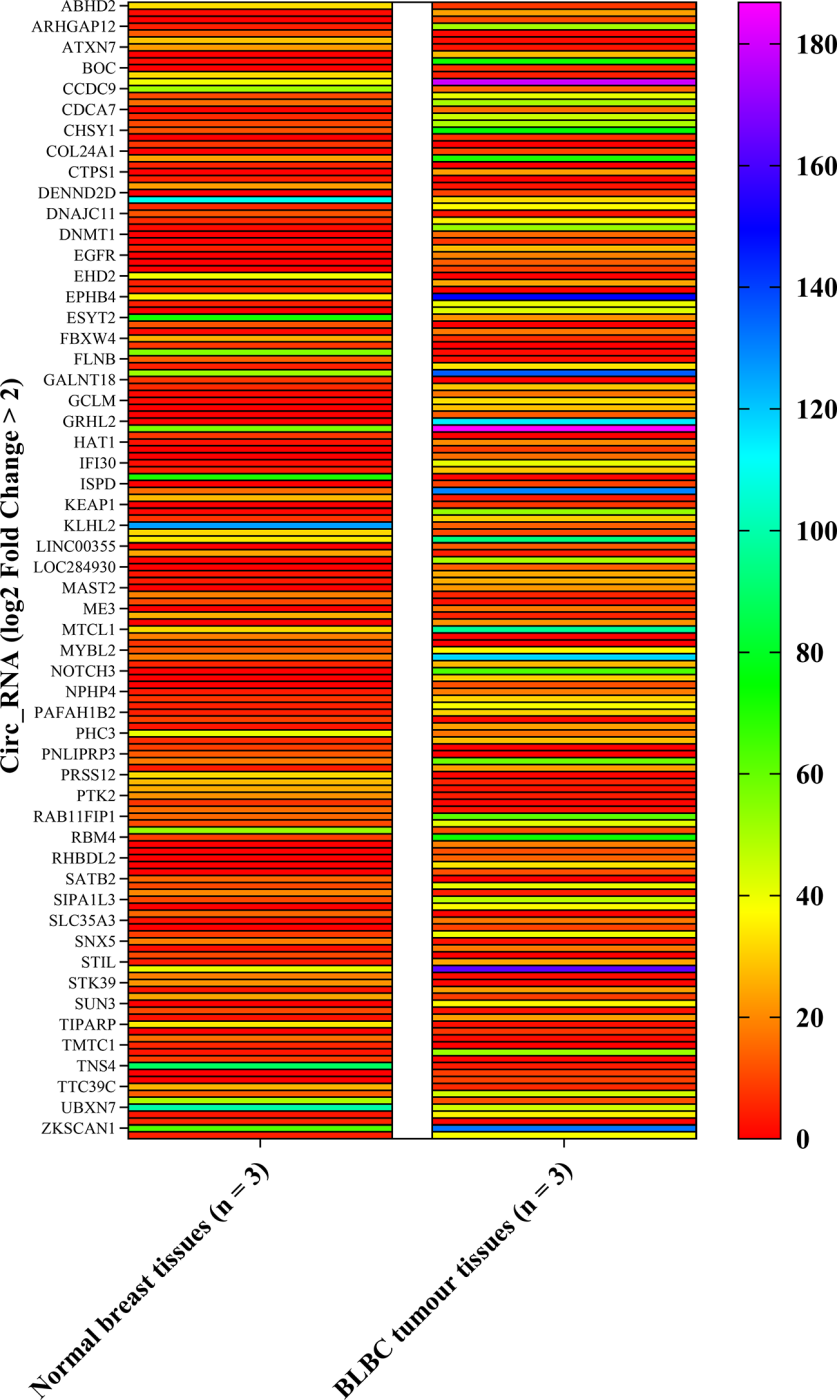
Heat map of circ_RNA array. The left row contains circ_RNAs. The column contains normal breast tissues and BLBC tumor tissues expression value. circ_NOTCH3, which the most upregulation log2FC times, was selected for further analysis. (log2FC>2). BLBC, basal-like breast carcinoma; FC, fold change; circ, circular.

The circ_RNAs expression profiles were first evaluated between BLBC tumor (n=3) and normal breast (n=3) tissue using circular RNA array. Array scanning data was extracted and a log2 fold change (log2FC)>2 was defined as the entry threshold. A total of 164 circ_RNAs were identified to be different between BLBC tumor tissues and the normal control, with 103 circ_RNAs upregulated (log2FC>2) and 61 downregulated (log2FC<−2; **Figure 1**). circ_NOTCH3, which had the highest log2FC value (7.8 times), was selected for further research.

### circ_NOTCH3 Is Highly Expressed in BLBC Tissues and Cell Lines

**Figure 2:**
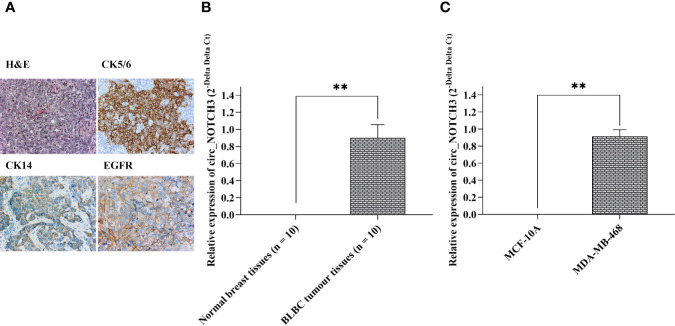
circ_NOTCH3 is overexpressed in BLBC. **(A)** BLBCs were characterized by a high histological grade, syncytial architecture with no glandular structures, regions of necrosis, and a prominent tumor infiltrating lymphocyte infiltrate. The tumor cells were negative for ER, PR and HER2 (triple-negative), and variably expressed CK5/6, CK14 and EGFR. The relative expression of circ_NOTCH3 was significantly higher in **(B)** BLBC tissues and **(C)** BLBC cell lines. **P < 0.01. BLBC, basal-like breast carcinoma; ER, estrogen receptor; PR, progesterone receptor; HER2, human epidermal growth factor receptor 2; EGFR, epidermal growth factor receptor; CK, cytokeratin; H&E, hematoxylin and eosin.

BLBC histopathological features were characterized by a high histological grade, pushing margins, syncytial architecture with no glandular structures, regions of necrosis and a prominent tumor-infiltrating lymphocyte infiltrate (**Figure 2A**). The tumors were negative for hormone receptors (ER and PR) and HER2 (triple-negative), and they variably expressed basal markers, such as cytokeratin (CK)5/6, CK14, EGFR (**Figure 2A**).

The relative expression of circ_NOTCH3 in 10 BLBC and paired normal breast tissues (adjacent to tumor from same patient) were detected by RT-qPCR, and it was shown to be significantly higher in BLBC than in normal breast tissue (**Figure 2B**; **P<0.01).

Next, the relative expression of circ_NOTCH3 in BLBCL cell lines (MDA-MB-468) and normal breast epithelial MCF-10A cell lines was further detected, and it was found to be significantly higher in MDA-MB-468 than in MCF10A cell lines (**Figure 2C**; **P<0.01).

### circ_NOTCH3 Promotes Proliferation and Inhibits Apoptosis in BLBC Cells

**Figure 3:**
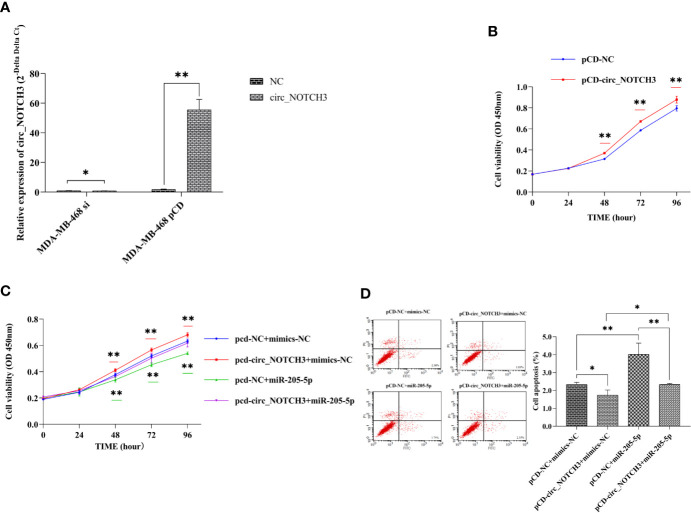
circ_NOTCH3 promotes proliferation and inhibits apoptosis. **(A)** The relative expression of circ_NOTCH3 was knocked down by siRNA and upregulated by pCD-circ_NOTCH3 transfection. **(B)** CCK-8 assay showed that the upregulation of circ_NOTCH3 expression markedly increased cell viability. **(C)** Cell viability was significantly increased by the overexpression of circ_NOTCH3, decreased by the overexpression of miR-205-5p, and restored by the simultaneous co-expression of circ_NOTCH3 and miR-205-5p. **(D)** The apoptosis assay showed that cell apoptosis was decreased by the overexpression of circ_NOTCH3 and significantly increased by the overexpression of miR-205-5p. Cell viability was decreased by the overexpression of miR-205-5p. Cell apoptosis was restored by the simultaneous co-expression of circ_NOTCH3 and miR-205-5p. *P < 0.05 and **P < 0.01. OD, optical density; circ, circulating; mi, micro; si, small interfering; CCK-8, cell counting kit-8.

To understand the role of circ_NOTCH3, si-circ_NOTCH3 and pCD-circ_NOTCH3 were transfected into MDA-MB-468 cells. The relative expression of circ_NOTCH3 was knocked down by siRNA transfection, and upregulated by pCD-RNA transfection (**Figure 3A**; *P<0.05, **P<0.01).

CCK-8 assay showed that the upregulation of circ_NOTCH3 expression markedly increased the cell viability of MDA-MB-468 cells (**Figure 3B**; **P<0.01). Following the transfection of pCD-circ_NOTCH3+mimics-NC into MDA-MB-468 cells, the cell viability was significantly increased, as compared with pCD-NC+mimics-NC (**Figure 3C**; **P<0.01). Transfecting pCD-NC+miR-205-5p into MDA-MB-468 cells, the cell viability was decreased (**Figure 3C**; **P<0.01). Cell viability was restored by the simultaneous co-expression of circ_NOTCH3 and miR-205-5p, as compared with the overexpression of circ_NOTCH3 and miR-205-5p (**Figure 3C**; *P<0.05 and **P<0.01, respectively).

The apoptosis assay showed that transfecting pCD-circ_NOTCH3+mimics-NC into MDA-MB-468 cells decreased cell apoptosis (%), as compared with the transfection of pCD-NC+mimics-NC (**Figure 3D**; *P<0.05). Following the transfection of pCD-NC+miR-205-5p into MDA-MB-468 cells, cell apoptosis (%) was significantly increased (**Figure 3D**; **P<0.01). Cell apoptosis (%) was restored by the simultaneous co-expression of circ_NOTCH3 and miR-205-5p, as compared with the overexpression of circ_NOTCH3 and miR-205-5p (**Figure 3D**; *P<0.05, **P<0.01, respectively).

### circ_NOTCH3 Promotes Migration and Invasion in BLBC Cells

**Figure 4:**
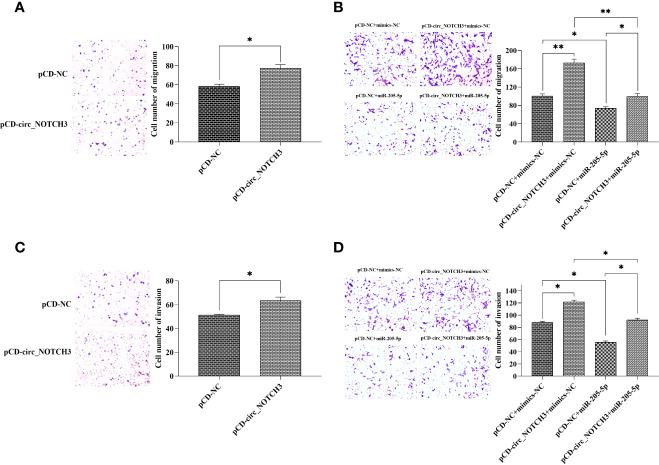
circ_NOTCH3 promotes migration and invasion. **(A)** Migration assay showed that the upregulation of circ_NOTCH3 increased the number of migrating cells. **(B)** The number of migrating cells was significantly increased by the overexpression of circ_NOTCH3, decreased by the overexpression of miR-205-5p, and restored by the simultaneous co-expression of circ_NOTCH3 and miR-205-5p. **(C)** The invasion assay showed that the number of invading cells was increased by the upregulation of circ_NOTCH3. **(D)** The number of invading cells was increased by the overexpression of circ_NOTCH3, decreased by the overexpression of miR-205-5p, and restored by the simultaneous co-expression of circ_NOTCH3 and miR-205-5p. *P < 0.05 and **P < 0.01. circ, circulating; mi, micro.

Transwell migration assay showed that the upregulation of the circ_NOTCH3 expression increased the number of MDA-MB-468 migrating cells (**Figure 4A**; *P<0.05). Following the transfection of pCD-circ_NOTCH3+mimics-NC into MDA-MB-468 cells, the number of migrating cells was significantly increased, as compared with pCD-NC+mimics-NC (**Figure 4B**; **P<0.01). Following the transfection of pCD-NC+miR-205-5p into MDA-MB-468 cells, the number of migrating cells was decreased (**Figure 4B**; *P<0.05). The number of migrating cells was restored by the simultaneous co-expression of circ_NOTCH3 and miR-205-5p, as compared with the overexpression of circ_NOTCH3 and miR-205-5p (**Figure 4B**; **P<0.01 and *P<0.05, respectively).

A Transwell invasion assay showed that the increase in circ_NOTCH3 expression increased the number of invading MDA-MB-468 cells (**Figure 4C**; *P<0.05). Following the transfection of pCD-circ_NOTCH3+mimics-NC into MDA-MB-468 cells, the number of invading cells was increased, as compared with pCD-NC+mimics-NC (**Figure 4D**; *P<0.05). Following the transfection of pCD-NC+miR-205-5p into MDA-MB-468 cells, the number of invading cells was decreased (**Figure 4D**; *P<0.05). The number of invading cells was restored by the simultaneous co-expression of circ_NOTCH3 and miR-205-5p, as compared with the overexpression of circ_NOTCH3 and miR-205-5p (**Figure 4D**; *P<0.05 and *P<0.05, respectively).

### circ_NOTCH3 Functions as a Molecular Sponge for MiR-205-5p in BLBC Cells

**Figure 5:**
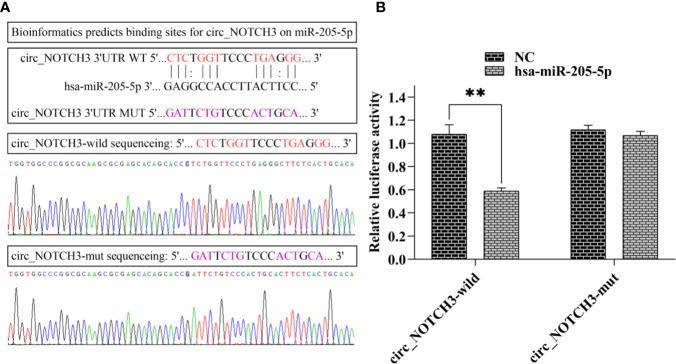
circ_NOTCH3 functions as a molecular sponge for miR-205-5p. **(A)** Putative binding site between circ_NOTCH3 and miR-205-5p, and sequences of circ_NOTCH3 wild and mutant type. **(B)** Dual-luciferase assay showed that the relative luciferase activity of circ_NOTCH3 was markedly decreased. **P < 0.01. circ, circulating; mi, micro.

Following bioinformatics prediction, a putative binding site was identified between circ_NOTCH3 and miR-205-5p (**Figure 5A**). Next, circ_NOTCH3 wild type and its mutant type were sequenced (**Figure 5A**). Dual-luciferase reporter gene assay was performed in order to confirm the speculation. The assay results showed that the relative luciferase activity of circ_NOTCH3 wild type was markedly decreased by miR-205-5p overexpression (**Figure 5B**; **P<0.01). By contrast, it had no effect on the relative luciferase activity of circ_NOTCH3 mutant type (**Figure 5B**).

### circ_NOTCH3 Binds to Mir-205-5p and Modulates Its Downstream KLF12 Expression

**Figure 6:**
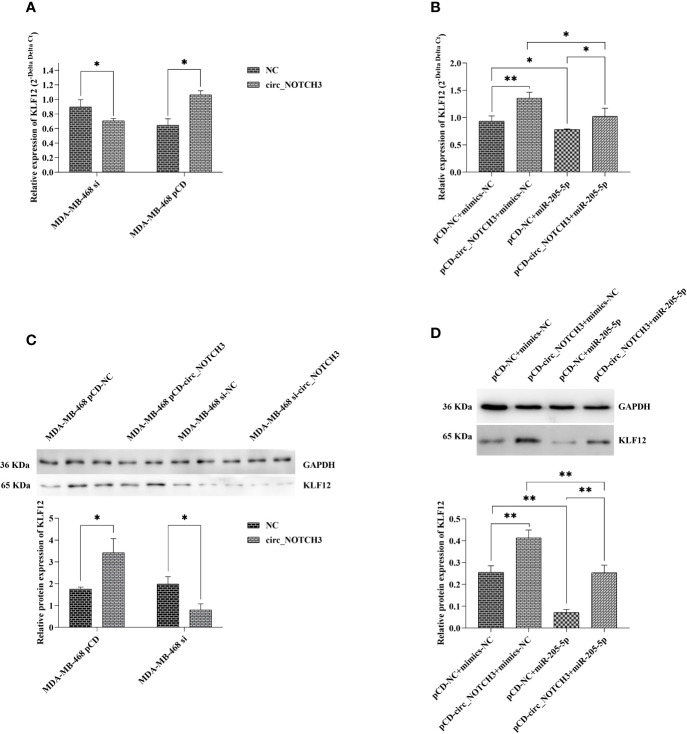
circ_NOTCH3 promotes the progression of BLBC by regulating KLF12 expression through sponges miR-205-5p. **(A)** RT-qPCR showed that the relative expression of KLF12 was knocked down by siRNA transfection and upregulated by pCD-RNA transfection. **(B)** The relative expression of KLF12 was markedly increased by the overexpression of circ_NOTCH3. The relative expression of KLF12 was decreased by the overexpression of miR-205-5p and restored by the simultaneous co-expression of circ_NOTCH3 and miR-205-5p. **(C)** Western blotting showed that the relative protein expression of KLF12 was knocked down by siRNA transfection and upregulated by pCD-RNA transfection. **(D)** The relative protein expression of KLF12 was significantly increased by the overexpression of circ_NOTCH3, markedly decreased by the overexpression of miR-205-5p, and restored by the simultaneous co-expression of circ_NOTCH3 and miR-205-5p. *P < 0.05 and **P < 0.01. circ, circulating; mi, micro; si, small interfering; BLBC, basal-like breast carcinoma.

RT-qPCR analysis showed that, following the transfection of si-circ_NOTCH3 and pCD-circ_NOTCH3 into MDA-MB-468 cells, the relative expression of KLF12 was knocked down by siRNA transfection, and upregulated by pCD-RNA transfection (**Figure 6A**; *P<0.05 and *P<0.05). Following the transfection of pCD-circ_NOTCH3+mimics-NC into MDA-MB-468 cells, the relative expression of KLF12 was markedly increased, as compared with pCD-NC+mimics-NC (**Figure 6B**; **P<0.01). Following the transfection of pCD-NC+miR-205-5p into MDA-MB-468 cells, the relative expression of KLF12 was decreased (**Figure 6B**; *P<0.05). The relative expression of KLF12 was restored by the simultaneous co-expression of circ_NOTCH3 and miR-205-5p, as compared with the overexpression of circ_NOTCH3 and miR-205-5p (**Figure 6B**; *P<0.05 and *P<0.05, respectively).

Western blotting showed that, following the transfection of si-circ_NOTCH3 and pCD-circ_NOTCH3 into MDA-MB-468 cells, the relative protein expression of KLF12 was knocked down by siRNA transfection, and upregulated by pCD-RNA transfection (**Figure 6C**; *P<0.05 and *P<0.05). Following the transfection of pCD-circ_NOTCH3+mimics-NC into MDA-MB-468 cells, the relative protein expression of KLF12 was significantly increased, as compared with pCD-NC+mimics-NC (**Figure 6D**; **P<0.01). Following the transfection of pCD-NC+miR-205-5p into MDA-MB-468 cells, the relative protein expression of KLF12 was markedly decreased (**Figure 6D**; **P<0.01). The relative protein expression of KLF12 was restored by the simultaneous co-expression of circ_NOTCH3 and miR-205-5p, as compared with the overexpression of circ_NOTCH3 and miR-205-5p (**Figure 6D**; **P<0.01 and **P*<*0.01, respectively).

## Discussion

circ_RNAs play an important role in cancer pathogenesis and progression (17, 21, 32). Due to intrinsic structure features, circ_RNAs lack 3’poly-A tails and 5’ end caps, and have long half-lives, which makes them resistant to regular mechanisms of linear RNA decay, so they can serve as efficient miRNA sponges (14, 16, 33, 34). Therefore, circ_RNAs could potentially be powerful biomarkers of cancer, given their long half-lives and resistance to common degradation pathways (35–37). However, direct circ_RNA research and its related miR/mRAN axis about BLBC were still lacking.

In the present study, the circ_RNA expression between BLBC tumor tissue and its NC was first screened using circ_RNAs array. Among the circ_RNAs with a differential expression, circ_NOTCH3 had the highest log2FC value (7.8 times), but its NC expression was 0. Furthermore, RT-qPCR results confirmed that circ_NOTCH3 had a significantly high expression in BLBC tissues and cell lines, and their NC expression was also 0. Given the circ_RNAs’ long half-lives and resistance to common degradation pathways, it supported that circ_NOTCH3 could be a powerful biomarker for BLBC.

Secondly, the role of circ_NOTCH3 in BLBC development and progression was evaluated. The siRNA and overexpression plasmid were transfected into MDA-MB-468 cells, and the relative expression of circ_NOTCH3 was knocked down and upregulated, respectively. The upregulation of circ_NOTCH3 significantly promoted proliferation vitality, inhibited cell apoptosis, and promoted cell migration and invasion. The upregulation of miR-205-5p markedly inhibited the proliferation vitality, promoted cell apoptosis, and inhibited cell migration and invasion. The simultaneous co-expression of circ_NOTCH3 and miR-205-5p restored proliferation vitality, and cell apoptosis, migration and invasion. In combination, these results suggested that circ_NOTCH3 played an important role in BCLC development and progression.

miRNAs are important post-transcriptional regulators of gene expression that act by direct base-pairing to target sites within UTRs of messenger RNAs (38, 39). circ_RNAs are highly stable and rich in miRNA reaction elements, making them highly efficient as miRNA sponges (12, 14, 17). In the present study, the most upregulated circ_RNA was found to be circ_NOTCH3, miR-205-5p, which targeted KLF12 (30, 31), bioinformatics analysis predicted its possible miR-205-5p binding sites, and dual-luciferase reporter assay finally supported the function of circ_NOTCH3 as a sponge for miR-205-5p. Further experiments showed that the relative expression of KLF12 was knocked down by siRNA transfection, and upregulated by the overexpression of circ_NOTCH3. The upregulation of circ_NOTCH3 expression led to an increase in the KLF12 mRNA and protein expression. P-regulation of the miR-205-5p expression led to a decrease in the KLF12 mRNA and protein expression. The simultaneously co-expression of circ_NOTCH3 and miR-205-5p restored the KLF12 mRNA and protein expression. This experimental evidence suggested that circ_NOTCH3 could be a protooncogene, function as a sponge and compete with miR-205-5p, modulating the KLF12 expression in BLBC.

Breast cancer has been a major public health problem worldwide among women (1–3), and thus breast cancer research has an important social significance. Recently, many related studies have shown that circ_RNF111 and circ_ABCB10 contribute to paclitaxel resistance in breast cancer (40, 41), circ_ZEB1 acts as an oncogene in triple-negative breast cancer (42), circ_TFF1 contributes to breast cancer progression (43), circ_0000526 blocks the progression of breast cancer (44), circulating circ_RNA hsa_circ_0001785 acts as a diagnostic biomarker for breast cancer (45), etc. In the present study, the major focus was on BLBC molecular subtype, and protooncogene circ_NOTCH3 was first identified to act as a sponge for miR-205-5p, which played a crucial role in its development and progression. The present results were also consistent with the more progression pathological features of BLBC. According to diagnostic molecular pathology, breast cancer is divided into surrogate subtype classification (6, 46), intrinsic subtype classification (4) and integrative cluster classification (47–49). However, only tissue from one subtype/cell line was analyzed. Considering the complexity of breast cancer classification, the present study revealed only the tip of the iceberg. The true number of circ_RNAs in breast cancer is almost certainly much larger. Due to the limitation of our clinical samples, we could not conduct more subtype research and related clinical treatment and prognosis studies, but aim to do so in the future.

In conclusion, the present experimental evidence suggested that circ_NOTCH3 functions as a sponge, competes with miR205-5p and modulates the KLF12 expression in BLBC. As a protooncogene, circ_NOTCH3 plays an important role in the development and progression of BLBC. circ_NOTCH3 can be a powerful biomarker for BLBC. Over time, it is highly likely that increasing evidence will emerge and enable us to better assess the clinical validity and utility of circ_NOTCH3 for the treatment of BLBC.

## Data Availability Statement

The original contributions presented in the study are publicly available. This data can be found here: NCBI SRA database, PRJNA663412 (https://www.ncbi.nlm.nih.gov/sra/PRJNA663412).

## Ethics Statement

The research protocol was officially approved by the Shanghai 6th People’s Hospital Jinshan Branch Medical Ethics Committee, and informed consent was obtained at the time of sample collection. The patients/participants provided their written informed consent to participate in this study. Written informed consent was obtained from the individual(s) for the publication of any potentially identifiable images or data included in this article.

## Author Contributions

BG conceived and designed the study. BG, QL, and H-ZZ reviewed and selected the cases. BG and QL acquired the data. BG analyzed and interpreted the data. BG wrote the manuscript. QL and H-ZZ reviewed the manuscript. QL, H-ZZ, and H-SY supervised the study. All authors contributed to the article and approved the submitted version.

## Funding

This work was supported by Shanghai Municipal Health Commission Clinical Research Project (Shanghai, China; grant no., 201840097), Shanghai Jinshan District Health Commission Youth Project (Shanghai, China; grant no., JSKJ-KTQN-2018-07), and Important Weak Subject Construction Project of Pudong Health and Family Planning Commission of Shanghai (Shanghai, China; grant no., PWZbr 2017-22).

## Conflict of Interest

The authors declare that the research was conducted in the absence of any commercial or financial relationships that could be construed as a potential conflict of interest.
